# Case Report and Literature Review: Interventional Management of Erythromelalgia

**DOI:** 10.31480/2330-4871/094

**Published:** 2019-07-29

**Authors:** Gregory Chinn, Zhonghui Guan

**Affiliations:** Department of Anesthesia and Perioperative Care, University of California San Francisco, San Francisco, California, USA

## Abstract

Erythromelalgia is a rare and very difficult to treat pain syndrome that usually presents as severe bilateral burning pain in the extremities. Here we present a case of a 34-year-old female with erythromelalgia who we treated successfully with a lumbar epidural infusion of ropivacaine and fentanyl. The patient had complete relief shortly after the epidural infusion, and she remained stable with only minor pain two weeks and nine months later. With this case, we have reviewed the interventional treatments of erythromelalgia. We suggest epidural infusion as the first line interventional management, followed by sympathetic block. Spinal cord stimulation can be considered if other interventional managements fail.

## Introduction

Erythromelalgia is a rare pain syndrome characterized by a bilateral burning sensation in the extremities that is often aggravated by physical activity or heat. The incidence is estimated as 1.3 per 100,000 people [1]. Erythromelalgia is very difficult to treat, with only a few reported cases of complete resolution. Commonly, patients resort to soaking the affected extremities in ice water as the only effective mediator of their pain. This prolonged exposure of the painful extremities to ice water can produce secondary damage caused by the reduced circulation from the low temperature.

Erythromelalgia is classified as either *primary* that occurs sporadically or is inherited, or *secondary* that is related to various autoimmune and myeloproliferative disorders. The etiology has remained elusive until the mapping of a familial form of erythromelalgia in the *SNA9a* gene, which encodes the Na_v_1.7 sodium channel as a gain-of-function mutation resulting in hyper-excitability in dorsal root ganglion (DRG) sensory neurons [2]. Further investigation of other familial erythromelalgia lineages has resulted in more than a dozen similar gain of function mutations of Na_v_1.7 sodium channel [2–4]. All these mutations act to enhance the excitability of DRG neurons by lowering the firing threshold. It has been hypothesized that given the similar phenotype, the non-genetic forms of erythromelalgia may be related to DRG neuronal hyper-excitability leading to neuro-humoral changes, particularly in distal limbs. Here we present a case of erythromelalgia successfully treated with a lumbar epidural of Ropivacaine/Fentanyl infusion. We also review current interventional treatments for erythromelalgia.

## Case Presentation

**Figure 1:**
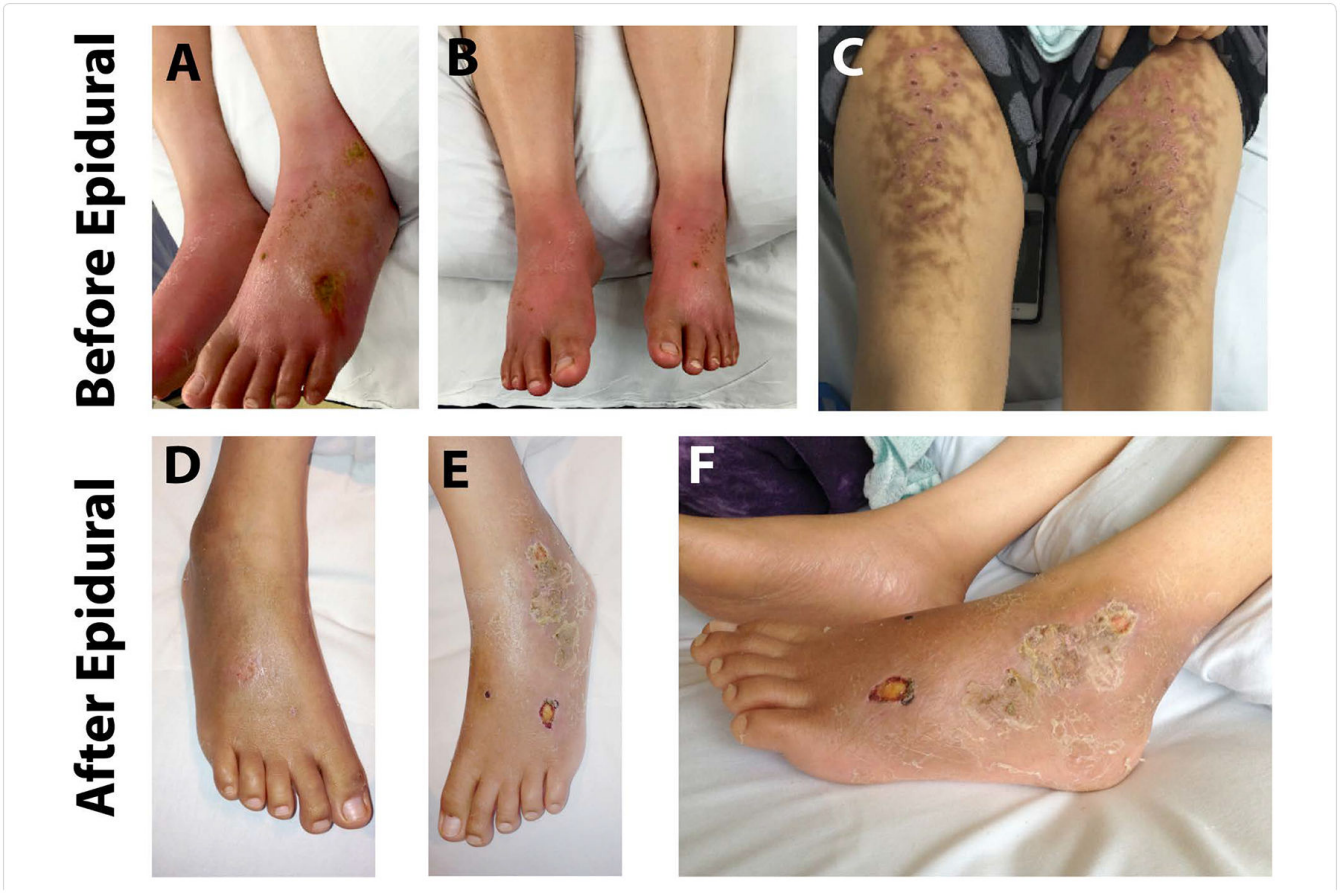
Skin findings before and after epidural treatment. **A./B.** Images of patient’s lower extremities at presentation. Note the ulcers on her feet from extended soaking in ice-water. **C.** Thighs have a rash consistent with livedo reticularis from heating pads to counteract the pain of soaking feet in ice-water. **D.-F.** Feet show dramatic decrease in erythema with healthy granulation tissue forming in bed of ulcers after treatment with epidural.

IRB approval was exempted from University of California San Francisco for this case report of patient treatment. A 34-year-old woman with a history of chronic constipation, recent episode of C. *difficile* colitis, and presumed alcoholic peripheral neuropathy presented to our medical center with severe burning pain in her feet after an exhaustive workup at an outside hospital. The pain was so severe that no medication was effective, including opioids, neuroleptics, and nonsteroidal anti-inflammatories (NSAIDs), and the only management that gave her pain relief was soaking her feet in ice baths. The pain was worse at night, but stayed at substantial severity during the daytime. She submerged her feet in ice water for 8–10 hours a day for pain relief, and she placed hot packs on her thighs to offset the cold discomfort from the ice-baths. On exam her feet were brightly erythematous and warm to touch bilaterally. Numerous ulcers were present on the dorsum of the feet (Figure 1a, and Figure 1b). She also had secondary tissue damage from both the hot packs on her thighs and the cold baths, resulting in a livedo reticularis rash (Figure 1c). She was evaluated by multiple specialists in our hospital, including neurology, vascular surgery, hospital medicine, and dermatology services, before the pain service was consulted. She had an extensive workup that included infectious, metabolic, rheumatologic, neurologic systems, with extensive imaging studies (Table 1). An electromyography (EMG) study showed an axonal neuropathy pattern. She was undergoing thiamine and folate supplementation with the thought that her pain might be related to alcohol consumption (she had periods of heavy binge drinking over the past 3 years, although none in the past 2 months).

Given her classic presentation for erythromelalgia, the pain service started the full dose of aspirin because significant subpopulation of erythromelalgia patients are responsive to aspirin^5^. However, she was unresponsive to aspirin and still reported her pain as 10/10. We subsequently placed a lumbar epidural and ran a continuous infusion of 0.0625% ropivacaine with 2 mcg/ml fentanyl at 12 ml/hour. Her pain score decreased to 0–1/10 almost immediately after the initiation of epidural infusion. We continued the epidural infusion over the next three days. The patient’s motor function was preserved and she was able to participate in physical therapy. On day four of epidural infusion we slowly weaned off the infusion, and the patient’s pain remained at 1–2/10 after the epidural infusion was discontinued. Physical examination showed that her feet were no longer brightly erythematous, but now were darkened with pigmentation. The feet were normothermic, the allodynia had been resolved, and the chronic ulcers in her feet started to heal with healthy granulation tissue noted (Figure 1d and Figure 1f). She was discharged home the next day, and the two-week follow-up visit in our chronic pain clinic documented that her pain remained at 0–2/10, and her functional status completely returned to her baseline. At a phone follow-up at nine months, she reported that her pain was still at 0–2/10, and she was maintaining her full activities of daily living. The CURES (Controlled Substance Utilization Review and Evaluation System) report confirms that she has not used any opioid since the treatment of epidural infusion.

## Discussion

Familial erythromelalgia is caused by mutation of sodium channel Na_v_1.7 [2] which leads to this unique constellation of symptoms. It is unlikely that our patient suffered from this genetic syndrome given her presentation in adulthood. Although we do not know the exact pathophysiology behind her symptoms, we postulate it was caused by abnormal function of DRG nociceptors with possible concomitant sympathetic nervous system dysfunction. The treatment with epidural ropivacaine served to suppress both pathways, and may have allowed a new homeostasis to occur. In addition, central sensitization in spinal cord, which could be inhibited as well by the epidural infusion of local anesthetics, might have also contributed to her pain syndrome. Whatever the mechanism, this case indicates that a standard epidural infusion should be considered as a modality to treat erythromelalgia in adults.

Medical treatment is the first line of therapies for the erythromelalgia. Commonly trialed medications include aspirin which may be effective in those with concurrent platelet dyscrasias caused by JAK-2 mutations [6]. Other commonly trialed medications include calcium channel blockers, sodium channel blockers, anticonvulsants, NSAIDs, and antidepressants [5]. However, many patients do not have a good response to medical treatment and invasive procedures for these patients may be justified.

In this case we have successfully treated an adult erythromelalgia patient with epidural infusion of 0.0625% ropivacaine with 2 mcg/ml fentanyl at 12 ml/hour for 3 days, and patient’s pain was well controlled even nine months after the treatment. While there are a few reported cases of epidural treatment that have led to good pain relief in pediatric patients [7–13], to our knowledge this one of a few adult case of erythromelalgia patient successfully managed with an epidural infusion [10,14].

Several invasive techniques have been described with varying degrees of success. Broadly they include providing sympathetic blockade or targeting central nervous system modulation. Peripheral nerve blocks, epidurals and sympathetic plexus blocks have been described with success in several cases. Although significantly different in technique, invasiveness and duration, they all likely act by inhibiting sympathetic outflow to the affected limbs. Here we summarize the relevant case reports and case series for these rare interventions (Table 1). One hypothesis for the origin of the pain of erythromelalgia is that it is related to regional tissue hypoxia secondary to abnormal microvascular circulation. Vasodilation associated with sympathectomy is thought to improve regional blood flow and restore normoxia to affected tissues^5^ which could account for the success of a variety of these techniques.

We found 8 case reports in the literature of epidural infusions to manage erythromelalgia patients from 6-year-old to 72-year-old (Table 1). Both lumbar and cervical epidural infusions have been successfully used to control pain in lower extremities and upper extremities, respectively. Epidural infusion duration varied from 2.5 days to 6 weeks, and they provided immediate and long-term (6 months to 4 years) benefits. In one case even 2 separated epidural boluses provided symptom control for over a year^14^. Although most cases, including ours, used the mixture of local anesthetic and opioids (fentanyl or morphine) for epidural treatment, epidural with local anesthetic alone without any opioid can provide similar benefit.

However, epidural is not always effective. In patients who do not respond to epidural, they may respond to sympathetic block^7^. We found 7 case reports in the literature treating erythromelalgia patients with lumbar sympathetic block from 2-month-old to 70-year-old (Table 1). Both short-term lumbar sympathetic block with local anesthetic, with or without steroid, and long-term lumbar sympathetic damage with pulse radio frequency or alcohol have been reported. No case report was found to treat erythromelalgia with stellate ganglion block, although it is very likely that stellate ganglion block might be able to provide symptom control of erythromelalgia in upper extremities.

More invasive interventional treatments like spinal cord neuromodulation has also been reported to treat erythromelalgia. There are 4 reports of trials of implanted spinal cord stimulators in patients from 15-year-old to 80-year-old [15–18] (Table 1). The leads were placed in the low thoracic region to manage symptoms of lower extremities, and in cervical region for upper extremity relief. The mechanism of action for this invasive modality is not completely understood and likely depends on the underlying cause of pain. For ischemic pain, spinal cord stimulation may inhibit sympathetic tone and improve oxygen delivery, like the sympathetic blocks, through vasodilation [19,20]. For neuropathic pain, spinal cord stimulators may relieve pain by modulating local neurotransmitter levels in the dorsal horns [19,20]. Similarly, targeting DRG neurons with stimulation has been reported as a treatment of erythromelalgia [16].

Even more invasive techniques have been described. One example is a 12 year-old with severe erythromelalgia symptoms that were leading to suicidality [21]. After infections forced the discontinuation of a spinal cord stimulation trial twice, bilateral thalamic electrodes were placed in the ventral posterolateral nuclei by a neurosurgeon. Continuous stimulation achieved improvement in pain, ablated suicidality, but had no effect on erythema. This invasive approach was inspired by an even more invasive approach described by a Soviet-era neurosurgeon of three Russian children who achieved complete resolution of erythromelalgia symptoms with stereotaxic ablation of the ventral posterolateral and the centromedian nuclei of the thalamus [22]. This irreversible, invasive, high-risk procedure performed in children raises many ethical questions, but may also offer insight into the mechanism of this disorder.

Erythromelalgia remains a challenging disorder to treat. Response to interventions will be highly dependent on the underlying cause of the disorder. Based on the risk benefit profile of all of the described invasive techniques we recommend first pursuing medical therapies first, ideally to be combined with cognitive and social support that are known to improve chronic pain [23]. An epidural infusion should be considered second line as the risks are low and relief can be significant and lasting in a variety of cases. If necessary, epidural can be done at the bedside without fluoroscopy. If epidural infusion does not offer good relief, or if it is not lasting, sympathetic blockade should be considered. Local anesthetic block of the sympathetic ganglion should be trialed to assess for patient response. With a good response, neurolysis can be achieved and may offer long-lasting relief. Unlike epidural, lumbar sympathetic block requires fluoroscopy and specific procedure room. Next, we suggest a trial of spinal cord stimulator given the expense and invasive nature of spinal cord modulation. Finally, with certain cases, especially those with contraindications to spinal cord stimulator, thalamic electrode implantation may be considered. While serious risks are associated with thalamic electrode implantation, erythromelalgia in some patients can be so severe and debilitating the benefits may justify the risks.

As the genetics and pathophysiology of erythromelalgia becomes better understood, targeted therapeutics will be developed which will hopefully improve quality of life for these patients. For those who have poor response to medications, invasive techniques will continue to play a role in threating this disorder.

## Figures and Tables

**Table 1:** 

Intervention	Patient	Details	Outcome	Citation
Epidural Infusion	17yo M	Failed medical tx including IV ketamine. LE peripheral nerve blocks ineffective. Ulcers infected required split thickness skin grafting; L3/4 epidural infusion (0.2% ropivacaine 10–12ml/hr)-2 weeks	Wounds healed, 6mo improvement of erythema and edema, discontinued opioids	Lee, 2017
	72yo F	EM and polycythemia vera with Jak2 mutation; started on alpha2-interferon and lumbar epidural infusion with PCEA (0.2% ropivacaine, 0.15Lidocaine and 5ug/mL fentanyl) for pain	Pain well controlled acutely, 4 year follow up mild symptoms	Li, 2016
	6yo M	Burning in hands>feet, bilateral axillary catheters placed- 0.25%bupivacaine 1wk; represented 2hrs later, sciatic catheters-placed then dislodged, then epidural catheter bupivacaine 0.25%, fentanyl 2mcg/ml- 6 weeks of treatment	2.5 yr follow up-resolution of pain, not taking medications	Harrison, 2003
	11yo M	Trialed on nalbuphine, pentoxifylline, nitroprusside in ICU without relief. L4–5 epidural infusion (0.125%bupivacaine, 5ug/mL fentanyl)- 1 wk	Acute pain control but long-term control with oral Mexiletine	Nathan, 2005
	28yo F	New diagnosis after workup as inpatient, discharged. L4–5 epidural placed in outpatient pain clinic. Bolus of 0.125% bupivacaine (10ml)- immediate improvement in pain, catheter left in place and patient returned following day, catheter was bolused again. On Day 3 symptoms were stable and catheter was removed.	1yr asymptomatic, off medication	Stricker, 2001
	12yo M & 17yo M	Both with significant upper extremity symptoms- cervical epidural (C7-T1) placed (bupivacaine 0.125–0.25%) at 5–10ml/hr (17 yo pt: 9 days, 12 yo pt:4 days then surgically placed epidural used for 37 days)	17 yo pt asymptomatic at 2yrs. 12 yo pt asymptomatic after 16 months	Rauk, 1996
	13yo M	Upper extremity pain, failed medical therapy and immunoglobulin tx, cervical epidural with bupivacaine and morphine for 2 wks	Improved erythema and edema. Significant atrophy of arms, required weeks of physical therapy to regain strength	Mohr, 1994
	15yo M	L3–4 epidural infusion (0.125%bupivacaine, fentanyl 1ug/ml) 12ml/hr for 2.5 days	Complete resolution at 1yr	D’Angelo, 1991
Sympathetic Block	22yo F	Lumbar sympathetic block- good relief-followed by pulse radio frequency tx of LSG	Improvement in VAS, continued on medication	Lee, 2016
	70yo F	Failed medical txs, bilateral LSG block with Ropivacaine 0.375%;	Improvement of pain, healing of ulcers after 6 wks	Cerci, 2013
	12yo F	Failed medical and caudal/epidural treatments, Bilateral LSG block with 1%lidocaine and triamcinolone	Moderate reduction in pain, LSG repeated, reduction in erythema, still soaking feet in water and waking at night	Bang, 2010
	18yo M	Failed medical treatments, admitted for intractable pain, LSG with alcohol	Two weeks post, discharged with improvement in symptoms-edema, pain, ulcers healing	Galemberti, 2009
	59yo M	LSG- alcohol ablation was placed after failing medical and epidural treatments	Immediate relief following unilateral, block, contralateral block pain relief for 9mo.	Seishima, 2000
	21yo F	Failed medical treatments, bilateral LSG, then Spinal block (8 times over 4 months)	Good relief after LSG and reduction in symptoms with intermittent spinal blocks	Takeda, 1989
	2 M, 1 F	10 days of consecutive LSG blocks alternative left and right	At 1 and 3 years, symptoms had not recurred	Buoncristiano, 1985
Spinal Cord Stimulator	20yr M	Two SCS generators, two cervical and two thoracic leads	Good pain relief, off all meds, return to normal fx	Eckman, 2017
	15yo F	T12-L1, two leads 2 V, 10 Hz, and 300-μs pulse width	Improvement in pressure related pain, feet remain erythematous and burning-remained on Mexiletine	Patel, 2015
	80yo F	T11 dual lead SCS	Good pain relief, dramatic reduction in opioids, return of functional status	Matzke, 2016
	69yo F	T9–10 quad lead SCS	Good relief after replacement of faulty generator	Graziotta, 1993
Brain Stimulation	12yo M	Had good relief with SCS which was removed for Staph infection twice, near suicidal after removal, doctors felt invasive nature was warranted. Thalamic electrode placed-bilateral ventral posterolateral nuclei (VPL)-210 microseconds, 100 Hz, 1.5 V right, and 2.9 V left	Not suicidal. Able to decrease medications and ice bath soaking. No change in erythema.	Delye, 2005
Thalamic nucleus ablation	11yo M, 13yo F, 15yo M	Varying degrees of severity of disease. Ablation of VPL with/without ablation of centromedian nucleus (CM)	Resolution of both pain and symptoms. One case, no resolution after unilateral ablation, but resolution after contralateral ablation Soviet era-raises ethical questions	Kandel, 1990
